# Construction and Experimental Validation of Embedded Potential Functions for Ta-Re Alloys

**DOI:** 10.3390/molecules29245963

**Published:** 2024-12-18

**Authors:** Haohao Miao, Xuehuan Xia, Yonghao Fu, Jing Yan, Lu Li, Hongzhong Cai, Xiao Wang, Chengling Wu, Zhaolin Zhan, Xian Wang, Zhentao Yuan

**Affiliations:** 1 Faculty of Materials Science and Engineering, Kunming University of Science and Technology, Kunming 650093, China; miaohao0304@163.com (H.M.); zhaolin_zkm@163.com (Z.Z.); 2 City College, Kunming University of Science and Technology, Kunming 650093, China; 3 Kunming Institute of Precious Metals, Kunming 650106, Chinaxy88@ipm.com.cn (X.W.)

**Keywords:** EAM potential functions, force matching, first-principles calculations, Ta-Re alloy

## Abstract

Ta/Re layered composite material is a high-temperature material composed of the refractory metal tantalum (Ta) as the matrix and high-melting-point, high-strength rhenium (Re) as the reinforcement layer. It holds significant potential for application in aerospace engine nozzles. Developing the Ta/Re potential function is crucial for understanding the diffusion behavior at the Ta/Re interface and elucidating the high-temperature strengthening and toughening mechanism of Ta/Re layered composites. In this paper, the embedded atom method (EAM) potential function for tantalum/rhenium binary alloys (Ta-Re alloys) is derived using the force-matching method and validated through first-principles calculations and experimental characterization. The results show that for the lattice constant of a bcc structure containing 54 atoms, surface formation energies per unit area of Ta-Re alloys obtained based on the potential function are 12.196 Å, E_100_ = 0.16 × 10^−2^ eV, E_110_ = 0.10 × 10^−2^ eV, and E_111_ = 0.08 × 10^−2^ eV, with error values of 0.015 Å, 0.04 × 10^−2^ eV, 0.02 × 10^−2^ eV, and 0.01 × 10^−2^ eV, respectively, compared with the calculations from first principles calculations. It is noteworthy that the errors in the average binding energies of Ta-rich (Ta_39Re20_, where the number of Ta atoms is 39 and Re atoms is 20) and Re-rich (Ta_20_Re_39_, where the number of Ta atoms is 20 and Re atoms is 39) cluster atoms, calculated by the potential function and first-principles methods, are only 1.64% to 1.98%. These results demonstrate the accuracy of the constructed EAM potential function. Based on this, three compositions of Ta-Re alloys (Ta_48_Re_6_, Ta_30_Re_24_, and Ta_6_Re_48_; the numerical subscripts represent the number of atoms of each corresponding element) were randomly synthesized, and a comparative analysis of their bulk moduli was conducted. The results revealed that the experimental values of the bulk modulus showed a decreasing and then an increasing tendency with the calculated values, which indicated that the potential function has a very good generalization ability. This study can provide theoretical guidance for the modulation of Ta/Re laminate composite properties.

## 1. Introduction

The nozzle is a critical component of the orbital control engine, exerting a significant influence on a spacecraft’s capability for precise velocity adjustments, orbital corrections, and attitude maintenance. It plays a pivotal role in ensuring the stable operation of the spacecraft. Given the extreme operational conditions it endures, the nozzle material must exhibit exceptional comprehensive properties, particularly high-temperature strength and oxidation resistance [1,2]. Refractory metals such as tungsten (W), molybdenum (Mo), tantalum (Ta), iridium (Ir), and rhenium (Re) have become primary materials for nozzle construction in thrust-controlled engines due to their high melting points and superior performance in high-temperature environments [3]. However, the rapid advancement of aerospace technology has led to increased demands for spacecraft in terms of flight distance, speed, and responsiveness. Traditional refractory metals are no longer adequate to meet these new requirements. Consequently, the advancement of a new generation of ultra-high-temperature materials with exceptional performance is crucial in the field of aerospace materials.

Rhenium (Re) is a rare metal known for its high melting point (3340 °C), high strength, creep resistance, and chemical stability. Ultramet, a U.S.-based company, utilized chemical vapor deposition (CVD) to prepare rhenium-based nozzle materials with service temperatures as high as 2200 °C. This innovation was successfully applied to the Hughes 601HP satellite, setting a new standard for nozzle material performance [4,5]. However, despite Re’s exceptional creep resistance and strength at high temperatures, it faces challenges, such as a high cost (USD 5163/kg), a high density (21.02 g/cm^3^), and complex preparation processes [6,7]. In contrast, tantalum and tantalum alloys possess high melting points, excellent strength, good processability, and lower density and cost, making them ideal structural materials for environments ranging from 1600 to 1800 °C [8]. For instance, alloys such as T-111, T-222, and ASTAR-811C, developed by NASA-affiliated research institutions, are widely used in alkali-metal corrosion-resistant pipelines, containers for radioactive materials, and rocket fuel containers [9].

Based on the superior properties of rhenium (Re) and tantalum (Ta), we designed a new Ta/Re layered composite material. This material leverages the cost-effectiveness and lightweight characteristics of tantalum, as well as the high strength of rhenium, and shows potential for applications in aerospace engine nozzle materials. During the research process, it was observed that the Ta/Re material, when exposed to high temperatures, exhibited interdiffusion of Ta and Re atoms at the Ta/Re interface, forming Ta-Re and Re-Ta solid solutions on either side of the interface, thereby resulting in alterations in the material’s high-temperature properties [10]. Consequently, investigating the diffusion behavior within the Ta/Re interface and studying the physical and chemical properties of the resulting solid solutions are crucial for enhancing the overall performance of Ta/Re layered composite materials [11,12]. However, since Ta (atomic number 73) and Re (atomic number 75) differ by only two atomic numbers and exist as a solid solution, conventional testing methods, such as transmission, struggle to distinguish their forms and mechanisms of action. Therefore, molecular dynamics simulations of the related interface reaction processes are required.

After over fifty years of development, molecular dynamics simulation (MD) has evolved into a robust tool for studying the diffusion behavior of both pure fluids and mixtures [13,14,15]. These simulations typically involve thousands of atoms, enabling detailed exploration of atomic-scale processes and offering insights into experimental phenomena at the microscopic level [16]. Consequently, MD serves as a valuable complement to experimental methods and theoretical models, providing a comprehensive understanding of diffusion mechanisms in various systems. Currently, molecular dynamics simulations are extensively employed in fields such as chemistry, biology, and materials science. Jamali et al. [17] conducted a thorough investigation into the finite size effects of MS diffusivities in binary mixtures. They performed EMD simulations of 200 LJ and nine molecular mixtures, showing that, similar to self-diffusivities, MS-diffusivities scale linearly with system size. Wei et al. [18] and Luo et al. [19] investigated the interface diffusion behavior of Mo-Ti and Fe-W interfaces using MD simulations, examining the effects of diffusion time, temperature, and interface orientation on diffusion. Notably, the potential function plays a pivotal role in molecular dynamics simulations. This mathematical function defines the interactions and energy exchanges among atoms, setting the force field and energy landscape within the simulated system. The choice of an appropriate potential function significantly affects the accuracy and reliability of simulation results. Unfortunately, the existing potential functions do not cover the Ta-Re system, making the construction of an appropriate potential function crucial for simulating its molecular dynamics processes and elucidating the interface diffusion mechanisms.

There are numerous types of potential function fitting methodologies. Among them, machine learning potentials have garnered significant attention in recent years due to their remarkable accuracy and ability to capture complex atomic interactions. Their flexibility in fitting large datasets and reproducing quantum-level precision makes them a powerful tool in materials modeling [20,21]. However, it is worth noting that machine learning potentials often lack strong physical interpretability, and their generalization ability across different systems may be limited. In contrast, traditional force field fitting methods offer a balance between accuracy and interpretability, providing well-defined physical parameters and ensuring broader applicability across diverse systems [22,23]. Therefore, employing the force field approach to construct the Ta-Re potential is a feasible solution.

To develop the Ta-Re embedded potential functions, a force-matching approach was employed. In this study, 90 different structures were considered, including fcc, bcc, sc, hcp, non-equilibrium fcc, low-index surfaces of bcc, fcc, sc, and hcp, and cluster types. Additionally, 9045 force measurements, 90 energy values, and 540 stress values were obtained through first-principles calculations. These data were then fitted using the force-matching method, and the Ta-Re potential function was derived using the Potfit software package. Finally, the accuracy of the potential functions was validated through both simulations and experimental methods. This study provides theoretical insight into the high-temperature strengthening and toughening mechanisms of Ta/Re layered composites and aids in controlling their material properties.

## 2. Results

To further validate the developed Ta Re alloy EAM potential model, we conducted multiple comparisons between first-principles calculations and experiments. These assessments covered the lattice constant, surface formation energies per unit area, defect formation energy in the single-crystalline state, and the bulk modulus for Ta-Re alloys with varying compositions of Ta and Re, representing a diverse range of structures. The test structures included bulk configurations, surfaces, and defective formations. Energy calculations were performed using the EAM potential model, which was not specifically fitted to all tested structures.

### 2.1. Bulk Structure

**Figure 1:**
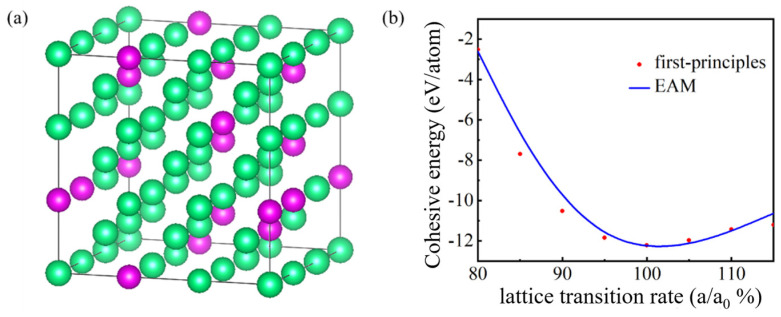
Structural model and cohesive energy of Ta-Re alloy: (**a**) the structural model of the face-centered cubic phase of the Ta-Re alloy with a Ta/Re ratio of 2:1. The green and violet spheres represent Ta and Re atoms, respectively; (**b**) the ratio of binding energy to the change in lattice constant, where “a” denotes the lattice constant after modification and “a_0_” represents the original lattice constant.

The functional capabilities of Ta-Re alloy crystals heavily depend on their structural attributes. Therefore, evaluating the predictive accuracy of our EAM potential function with respect to the behavior of Ta-Re alloys is of utmost importance. To achieve this, we constructed a 3 × 3 × 3 bcc supercell containing 54 atoms, with Ta as the primary element and Re as the alloying element. The distribution of Ta and Re atoms adhered to a 2:1 Ta/Re ratio, as shown in Figure 1a. With respect to the crystal structure, we expect a lattice constant of 12.196 Å for the bcc supercell, which closely matches the value of 12.211 Å obtained from first-principles calculations. We evaluated the cohesive energy of Ta-Re alloy under different strain states through experimental calculations. The cohesive energy values, plotted against the lattice transition rate, are shown in Figure 1b. It is satisfactory that the cohesive energy curve calculated by our EAM potential function has a high degree of agreement with the cohesive energy curve calculated using first principles. This alignment indicates the successful representation of atomic interactions by our EAM potential function during compression, equilibrium, and expansion in Ta-Re crystal structures of the binary alloy.

### 2.2. Surface Energy

**Figure 2:**
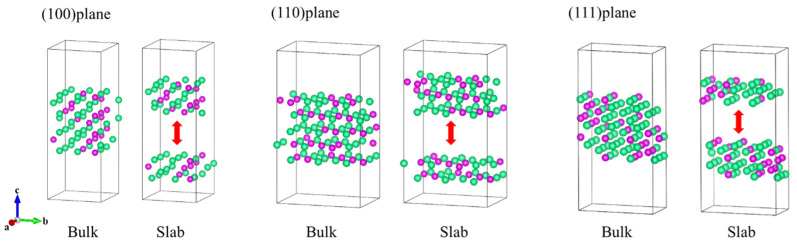
Surface models of three different Ta-Re alloys. Red arrows indicate the location of the cuts. Among them, the green and purple spheres are Ta and Re atoms, respectively.

The surface formation energy per unit area was calculated using the EAM potential function, and the results were compared with those from first-principles calculations. Three typical low-index planes, (100), (110), and (111), were constructed, as shown in Figure 2. Each surface model consists of Ta and Re atoms in a 2:1 ratio, with Ta and Re atoms randomly arranged both in the bulk and on the surface. Based on the definition of surface energy per unit area, we performed the calculations and analyzed the results.

(1) $$E_{surf}=\frac{E_{slab}-E_{bulk}}{2S}$$

Here, *E_slab_* and *E_bulk_* represent the total energies of the slab and bulk structures shown in Figure 2, respectively, while *S* denotes the surface area. We calculated the surface formation energy per unit area for three different surface types. The values represent the energy required to create a unit area of surface on the respective crystallographic planes. The smaller the surface energy value, the more stable the surface.

As shown in Table 1, the surface formation energy per unit area predicted by the EAM potential function is lower than that obtained from first-principles calculations. The surface energy errors per unit area for the (100), (110), and (111) planes of Ta-Re alloys calculated using the fitted EAM potential and first-principles calculations are 0.04 × 10^−2^ eV, 0.02 × 10^−2^ eV, and 0.01 × 10^−2^ eV, respectively. This discrepancy arises because the EAM potential is optimized for large-scale simulations and cannot fully capture electronic local effects. At the surface, the EAM potential slightly underestimates the effect of dangling (unsaturated) bonds, which results in a lower surface energy. Nevertheless, the EAM potential offers a reasonable approximation of the surface properties of Ta-Re alloys [24].

### 2.3. Monovacancy Defects

**Figure 3:**
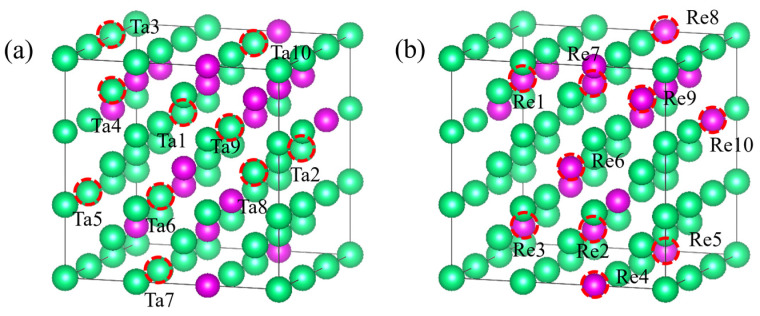
Location of defects in Ta-Re bulk. (**a**) Ta atomic vacancies, (**b**) Re atomic vacancies. Among them, the green and purple spheres are Ta and Re atoms, respectively.

In real materials, point defects are commonly present and can significantly affect various physical and chemical properties. This study specifically examines the monovacancy defect as a representative case. A 3 × 3 × 3 bcc supercell was constructed with Ta and Re atoms randomly distributed in a 2:1 ratio. Figure 3 depicts a randomly generated monovacancy site within the bulk phase of a supercell consisting of 54 atoms. The formation energy of the monovacancy defect is calculated using the following formula:

(2) $$E_{defect}=E_{atom}+E_{Monovacancy}-E_{perfect}$$

where $E_{Monovacancy}$ and $E_{perfect}$ represent the total energies of Ta-Re alloys with and without monovacancy defects, respectively, and $E_{atom}$ is the total energy of a free Ta or Re atom.

The formation energies of monovacancy defects for the studied configurations, calculated using our EAM potential function, are presented alongside those obtained from first-principles calculations in Table 2. In this study, the monovacancy defect formation energies calculated using our fitted EAM potential function show some discrepancies compared to the results from first-principles calculations. This discrepancy may arise from the limitations of the EAM potential function in describing local defects, particularly its inability to accurately capture electronic structure effects such as defect-induced electron rearrangements. Additionally, due to the differences in electronic structure between Ta and Re atoms, the model may describe their defects differently. However, despite these discrepancies, the EAM model provides a reasonable description of the overall trends in large-scale simulations and remains reliable for describing the macroscopic properties of Ta-Re alloys.

### 2.4. Cluster Testing

**Figure 4:**
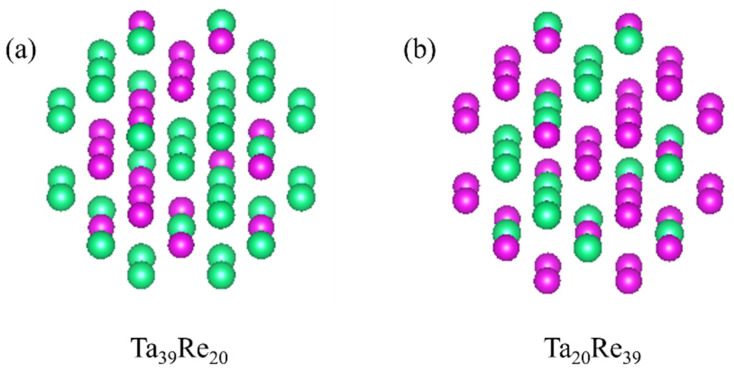
Cluster structures with different Ta-Re contents: (**a**) Ta_39_Re_20_, (**b**) Ta_20_Re_39_. Among them, the green and purple spheres are Ta and Re atoms, respectively.

Finally, we evaluated the transferability of the EAM potential function by performing test calculations on Ta-rich (Ta_39_Re_20_) and Re-rich (Ta_20_Re_39_) atom clusters. The structures of these clusters are shown in Figure 4, and Table 3 presents the atomic mean binding energies calculated using both first-principles methods and the EAM potential function. The results indicate that the binding energies predicted by the EAM potential are slightly higher than those from first-principles calculations, Ta-rich (Ta_39_Re_20_) and Re-rich (Ta_20_Re_39_), with errors ranging from 1.64% to 1.98%. This is because the EAM potential in this study is an empirical model fitted using the force-matching method, which does not fully account for the details of electronic structure and surface effects. In contrast, first-principles calculations consider a more comprehensive range of electronic and interatomic interactions, resulting in more accurate binding energy values [25].

## 3. Experimental Testing and Analysis

### 3.1. XRD Patterns of Ta-Re Alloy Specimens

**Figure 5:**
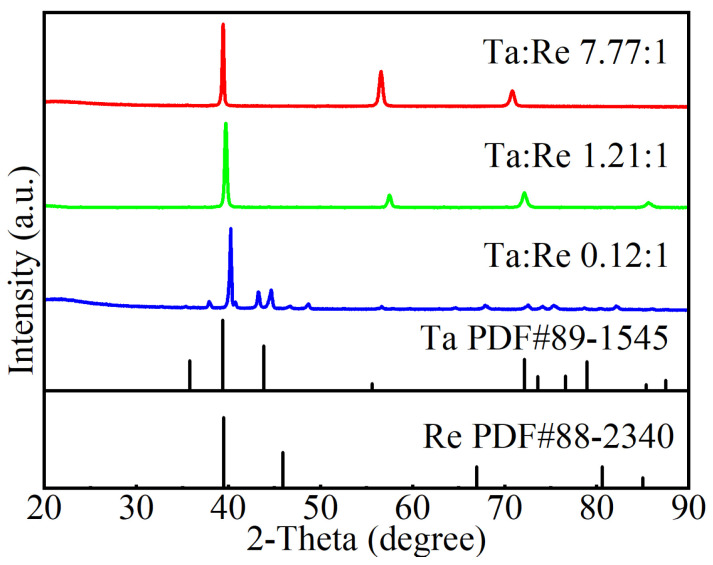
XRD patterns of mass ratios Ta-Re alloy specimens.

To precisely characterize the composition and crystal structure of Ta-Re alloys, we performed X-ray diffraction (XRD) analyses on the prepared alloy samples, as illustrated in Figure 5. The XRD spectra of Ta-Re alloys, as depicted in Figure 5, closely match the standard PDF cards PDF #88-2340 (Re) and PDF #891545 (Ta). Notably, the XRD pattern showed no evidence of intermetallic phases.

When the mass ratios of Ta to Re are 7.77:1 and 1.21:1, respectively, the Ta-Re alloy exhibits a solid solution structure in which tantalum (Ta) serves as the matrix and rhenium (Re) functions as the solute element. It is important to note that, in this context, the solubility of Re in Ta does not exceed its maximum solubility limit [26]. As the Re content further increases (Ta:Re = 0.12:1), the microstructure of Ta-Re alloys undergoes a transformation, with rhenium emerging as the primary phase and tantalum as the secondary phase. This transition results from the relatively low solubility of tantalum in rhenium (2735 °C, 5 wt%) [27]. Excess tantalum in the alloy precipitates as the temperature decreases, resulting in the coexistence of both Re and Ta phases.

### 3.2. Microstructure of Ta-Re Alloy Specimens

**Figure 6:**
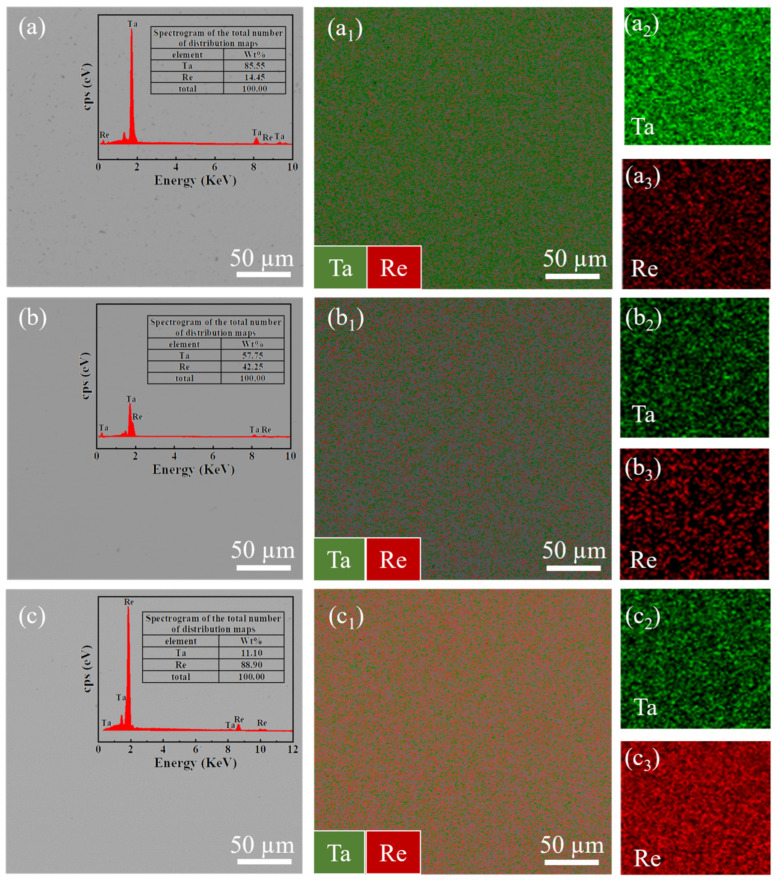
Microstructure and elemental distribution maps of Ta-Re alloy specimens: (**a**) mass ratio Ta:Re = 7.77:1, (**b**) mass ratio Ta:Re = 1.21:1, (**c**) mass ratio Ta:Re = 0.12:1. The subsubfigures represents the elemental distribution of Ta and Re in Ta-Re alloys of different compositions.

The microstructural morphology and elemental distribution of Ta-Re alloys with varying Ta and Re contents are illustrated in Figure 6. The figure demonstrates a notably uniform distribution of Ta and Re elements throughout the alloy’s microstructure, with no discernible segregation. The Re contents in alloys with Ta:Re ratios of 7.77:1 (Figure 6a), 1.21:1 (Figure 6b), and 0.12:1 (Figure 6c) are 14.45 wt%, 42.25 wt%, and 88.90 wt%, respectively, closely aligning with the targeted design specifications. This observation confirms that the prepared specimens fulfill the criteria for experimental validation.

The variations in the matrix, elemental distribution, and content of Ta-Re alloys become pronounced as the Ta content increases. At a Ta:Re ratio of 7.77:1, the alloy is Ta-based, with Re uniformly dispersed within the Ta matrix. As the Re content increases, approaching a Ta:Re ratio of 1.21:1, the Ta and Re contents in the alloy’s microstructure become comparable, at 57.75 wt% and 42.25 wt%, respectively. It is important to note that, even under these conditions, the alloy matrix remains predominantly composed of Ta [28]. Further increasing the Re content, corresponding to a Ta:Re ratio of 0.12:1, induces a transition in the microstructure of the Ta-Re alloy, converting it into a Re-based solid solution, where Re acts as the matrix and Ta as the solute element.

### 3.3. Bulk Elastic Modulus

**Figure 7:**
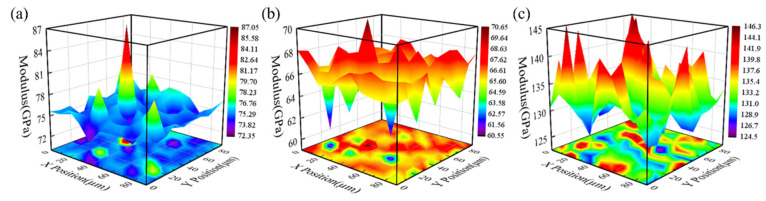
The bulk elastic moduli for different Ta: Re mass ratios (**a**) 7.77:1; (**b**) 1.21:1; (**c**) 0.12:1.

To verify the accuracy of the constructed EAM potential function, we obtained experimental bulk modulus data for Ta-Re alloys with varying compositions through 10 × 10 dot matrix (shown in Supplementary Figure S1) nanoindentation experiments using the iMicro device. The bulk modulus values of these alloys were then calculated using the EAM potential function, as shown in Figure 7 and Table 4.

As shown in Figure 7, the bulk modulus values of each Ta-Re alloy are relatively uniform, with localized regions of “raised” or “depressed” values due to solid solution strengthening or crystal structure defects. Overall, the bulk modulus first decreases and then increases as the Re content increases. Table 4 shows that the measured bulk moduli of alloys with Ta:Re mass ratios of 7.77:1, 1.21:1, and 0.12:1 are 77.31 GPa, 66.67 GPa, and 145.27 GPa, respectively, while the calculated values are 61.43 GPa, 54.59 GPa, and 114.96 GPa, respectively. Notably, the trend of the bulk modulus with Re content is consistent across both methods, providing partial validation of the constructed potential function. Table 4 also presents the differences between the bulk modulus values calculated using the EAM potential function and those obtained from experimental tests. Their error percentages are 21%, 18.1%, and 21%, respectively. These discrepancies can be attributed to differences in the conditions of molecular dynamics simulations and experimental tests, as well as factors such as the presence of lattice defects in the specimens, which were not accounted for in the simulations [29].

## 4. Fitting Method and Experimental Details

### 4.1. EAM Potentials

The embedded atom method (EAM) [30] is a computational model primarily used for analyzing atomic interactions within metal systems, focusing on the structure and interplay of atoms. This method operates on the principle of simulating atomic interactions by characterizing the embedding energy associated with electrons around each individual atom. The total energy of an alloy system can be expressed concisely in a generalized form.

(3) $$E_{tot}=\sum_{i}E_{i}$$

(4) $$E_{i}=F_{\alpha }\left(n_{i}\right)+\frac{1}{2}\sum_{\begin{array}{c} i,j\\ i\neq j \end{array}}\Phi_{\alpha \beta }\left(r_{ij}\right)$$

Among them, *E_i_* represents the total potential energy of the system, which is the linear sum of the individual potential energies of all embedded atoms in the system. Furthermore, *i* and *j*, respectively, represent embedded atoms and adjacent atoms within the system. The types of embedded atoms and adjacent atoms are represented by *α* and *β*, respectively.

In Equation (4), the initial component denotes the embedding energy of the i atom, illustrating the interaction between the atom i and the charge density of its background electrons. A reference is provided for the initial formulation of the embedding function $F\left(n\right)$ [31]:

(5) $$F\left(n\right)=F_{0}\left[\frac{q}{q-p}{\left(\frac{n}{n_{e}}\right)}^{p}-\frac{p}{q-p}\left(\frac{n}{n_{e}}\right)\right]+F_{1}\frac{n}{n_{e}}$$

The formulation is derived from the more general equation proposed by Rose et al. [32]. Real values are taken for the parameters p and q, while $n_{e}$ represents the equilibrium density. Due to numerical instability with our optimization algorithms in the original form, we substitute p with q and set $n_{e}$ = 1. Consequently, Equation (5) simplifies to the following expression.

(6) $$F\left(n\right)=F_{0}\left[1-\gamma lnn\right]n^{\gamma }+F_{1}n$$

The equation involves three free parameters, $F_{0}$, $F_{1}$, and γ. In our proposed model, we utilize the simplified form presented in Equation (6).

In Equation (4), $n_{i}$ represents the charge density of the host. In this calculation, the electron density contributions from atoms within a cutoff radius *r_cut_* are summed linearly, excluding the embedding atom itself.

(7) $$n_{i}=\sum_{j\neq i}\rho_{\beta }\left(r_{ij}\right)$$

It is important to note that this charge density is purely empirical and does not correspond to the actual physical density. Subsequently, the electron transfer function $\rho_{\beta }\left(r_{ij}\right)$ can be expressed [33].

(8) $$\rho_{\beta }(r)=\Psi \left(\frac{r-r_{c}}{h}\right)\frac{1+a_{1}cos\left(\alpha r+\varphi \right)}{r^{\beta }}$$

We can see from the transfer function that this oscillation type is characterized by four free parameters, *a*, *a*_1_, *j*, and *β*. Among them, *a*_1_ represents the amplitude of this type of oscillation, while h is used to smooth the potential. It is worth noting that *a*, *a*_1_, and *β* also correspond to the wave vector, initial phase, and attenuation factor of the wave function, respectively.

As shown in Equation (4), the second term represents the pair potential between atoms i and j separated by a distance *r_ij_*. For our test calculations, to verify the accuracy of the model, we selected the Empirical Oscillating Pair Potential (EOPP) [34], the Classical Morse potential (CM) [35], and the Lennard Jones (LJ) potential [36] for testing and calculation. Our findings indicate that the EOPP demonstrates significant flexibility in describing atomic interactions within the Ta-Re system. Consequently, we adopt the function of the pair potentials with oscillations, expressed as follows:

(9) $$\Phi (r)=\Psi \left(\frac{r-r_{c}}{h}\right)\left[\frac{c_{1}}{r^{\eta_{1}}}+\frac{c_{2}}{r^{\eta_{2}}}\cos\left(kr+\varphi \right)\right]$$

In the provided formulation, short-range repulsion is governed by the parameters *c*_1_ and *η*_1_, while *c*_2_ and *η*_2_ are the key to maintaining oscillation with *k* as the characteristic frequency. It is worth noting that this oscillation, based on empirical definitions, has been widely applied in the study of ternary alloy systems and other complex metal alloy systems [37,38]. In Equations (8) and (9), the cutoff function Ψ(x) is defined [37].

(10) $$\Psi (x)=\frac{x^{4}}{1+x^{4}}$$

where Ψ(x) ≡ 0 for x ≥ 0 and Ψ(*x*) is defined for *x* < 0. This function ensures that at the distance cutoff $r_{c}$, the potential function and its derivatives up to the second order smoothly approach zero. Both h and $r_{c}$ act as global parameters with uniform values across all functions. The cutoff radius $r_{c}$ in our model is uniformly set to 6.0 Å throughout the fitting process.

All potential functions are employed in their analytical forms. The atomic interactions in a binary tantalum/rhenium (Ta-Re) alloy system are characterized by a total of seven analytical functions. These include three pair potential functions ($\Phi_{Ta-Ta}$, $\Phi_{Ta-Re}$, and $\Phi_{Re-Re}$), two electron transfer functions ($r_{Ta}$ and $r_{Re}$), and two embedding functions ($F_{Ta}$ and $F_{Re}$). There are a total of 17 free parameters included in this potential function. Among them, the electron transfer function (TF) has four free parameters, the electron potential function (PPF) has six free parameters, the embedding function (EF) has six free parameters, and, finally, there is a global parameter *h* as a whole. This potential function serves as the foundation for the analysis. The above parameters can be obtained by fitting the potential energy surfaces of different structures of the studied Ta Re alloy system.

### 4.2. Target Systems for Fitting

**Figure 8:**
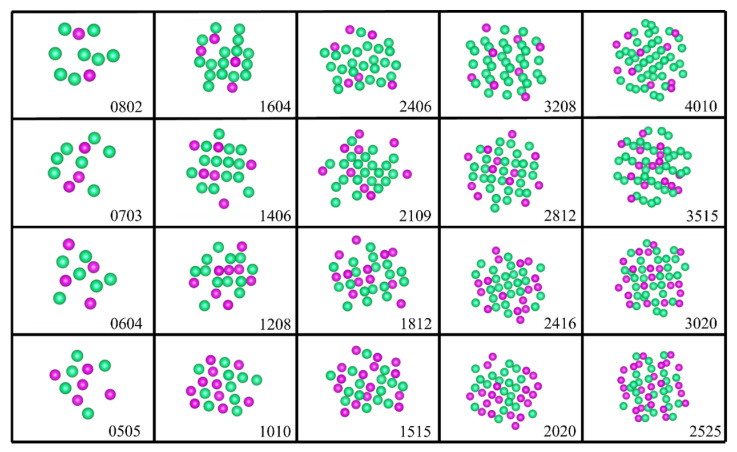
Partial cluster reference structures. The lower right corner of each cluster displays four numbers that indicate the respective quantities of tantalum (Ta) and rhenium (Re) atoms. For instance, “0802” signifies 8 Ta atoms and 2 Re atoms. Among them, green spheres represent Ta atoms, while purple spheres denote Re atoms.

**Figure 9:**
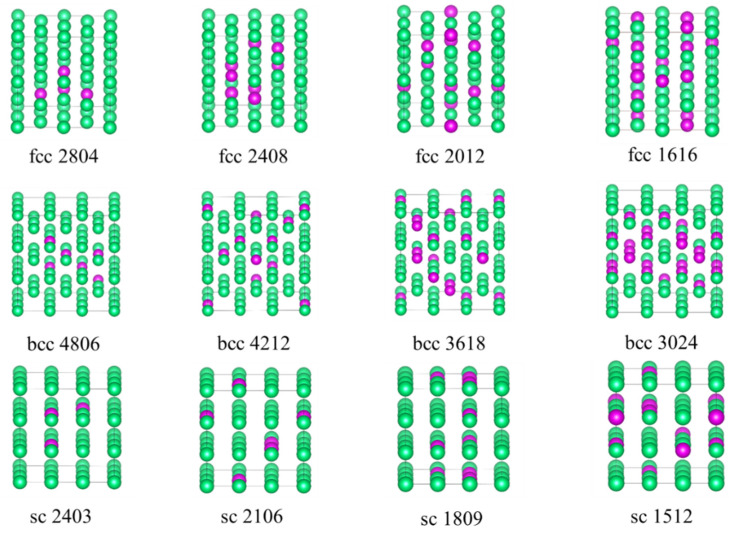
The crystals of fcc, bcc, and sc have their partial reference structures depicted. The four numbers below each structure represent the numbers of atoms in Ta and Re, respectively. Among them, the purple balls and green balls are Re atoms and Ta atoms, respectively.

**Figure 10:**
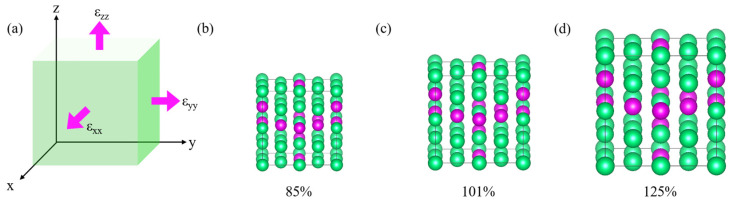
Schematic diagram of structural relaxation: (**a**) strain schematic with applied strain for some of the reference structures: (**b**) 85%, (**c**) 101%, and (**d**) 125%. Among them, the Ta/Re ratio is 20/12. the green and purple spheres are the Ta and Re atoms, respectively.

During the fitting of the EAM potential function for Ta-Re alloys, a large number of reference configurations were thoroughly evaluated to enhance the transferability of the EAM potential function. This careful evaluation ensures that the model accurately represents the alloy’s behavior under various conditions. The configurations include clusters, face-centered cubic (fcc), body-centered cubic (bcc), simple cubic (sc), hexagonal crystals, and non-equilibrium structures, as shown in Figure 8, Figure 9 and Figure 10 and Supplementary Figures S2–S4. To ensure precision, we performed full relaxation of most reference configurations using first-principles calculations, with a maximum force of less than 0.01 eVÅ^−1^ per atom.

The clusters exhibit a substantial specific surface area and unique electronic properties. Their intricate electronic and structural characteristics are influenced by several factors, including size and composition. In our existing database, we have curated five clusters of varying sizes, comprising 10, 20, 30, 40, and 50 atoms, respectively. Moreover, each cluster size encompasses different proportions of Re atoms, specifically 20%, 30%, 40%, and 50%. The reference structure is depicted in Figure 8.

To enhance the generalization ability of the potential function, it is essential to consider both equilibrium and non-equilibrium structures, particularly in temperature simulations of complex systems that involve local perturbations, segregation, clustering, and precipitation.

For equilibrium structures, we constructed a 32-atom 2 × 2 × 2 supercell to represent the periodic face-centered cubic (fcc) phase and selected seven Ta_32-x_Re_x_ alloy structures with varying compositions (x = 4, 8, 12, 16, 20, 24, 28). Similarly, for the body-centered cubic (bcc) phase, a 3 × 3 × 3 supercell containing 54 atoms was constructed, and we identified eight Ta_54-x_Re_x_ alloy structures (x = 6, 12, 18, 24, 30, 36, 42, 48). For the simple cubic (sc) phase, a 3 × 3 × 3 supercell containing 27 atoms was employed, and eight Ta_27-x_Re_x_ alloy structures (x = 3, 6, 9, 12, 15, 18, 21, 24) were selected. Partial reference structures for the face-centered cubic (fcc), body-centered cubic (bcc), and simple cubic (sc) crystals are illustrated in Figure 9. The added non-equilibrium structure is shown in Table 5.

**Figure 11:**
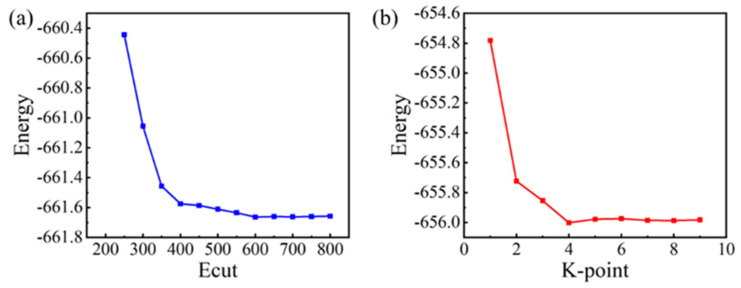
The convergence test of cutoff energy and k-point: (**a**) cutoff energy, (**b**) k-point.

The reference database includes 90 structures, comprising 20 cluster structures, 7 face-centered cubic (fcc), 8 body-centered cubic (bcc), 8 simple cubic (sc), and 47 non-equilibrium structures. These configurations were carefully selected to provide comprehensive coverage of the Ta-Re alloy system. The database contains 9045 force measurements, 90 cohesive energies, and 540 stress values, all derived from first-principles calculations performed using the Projector Augmented Wave (PAW) method [39,40] within the Vienna Ab Initio Simulation Package (VASP) code [41,42]. The Generalized Gradient Approximation (GGA) was applied for the exchange–correlation functional, specifically using the PW91 functional [43]. In the electronic structure calculations, the valence electrons were treated as Ta: 5d^3^6s^2^ and Re: 5p^6^5d^5^6s^2^. According to the cutoff energy and k-point convergence tests based on the Monkhorst–Pack method shown in Figure 11, a set of plane-wave basis vectors with a cutoff energy of 650 eV is used to expand the electronic wavefunction, and the k-point grid is set to 7 × 7 × 7, which meets the accuracy requirements. Since the Ta-Re system is non-magnetic, spin polarization effects were not considered in any of the calculations. Additionally, a 15 Å vacuum region was incorporated along the x, y, and z directions to prevent unwanted interactions between clusters during the simulations.

### 4.3. Fitting Procedure

In this paper, we have fitted the EAM potential function for Ta-Re alloys using the force-matching method [44] and the Potfit package [45,46] developed by Brommer et al. During the fitting process, the results from first-principles calculations were used as a crucial reference for refining the free parameters of the potential function. Additionally, the ranges of the electron density function *ρ_β_*(*r*) and the electron potential function *Φ_αβ_*(*r*) were defined between the minimum distance *r_min_* and the cutoff distance *r_c_*. The electron density function determines the effective range of the embedding function *F_α_*(*n*). The minimum interatomic distance in a given configuration corresponds to the original value of *r_min_*, while the cutoff distance of 6.0 Å encompasses interactions between atoms and their fourth-nearest neighbors in the equilibrium system.

To achieve accurate parameter estimation, we employ two distinct optimization algorithms to fit our analytical function. Both algorithms are specifically designed to minimize the sum of squares, expressed as follows:

(11) $$Z=\sum \frac{{\left(f-f_{0}\right)}^{2}}{f_{0}^{2}+\varepsilon_{j}}+\sum w_{E}\left(\frac{{\left(E-E_{0}\right)}^{2}}{E_{0}^{2}+\varepsilon_{k}}\right)+\sum w_{s}\left(\frac{{\left(S-S_{0}\right)}^{2}}{S_{0}^{2}+\varepsilon_{l}}\right)$$

In this context, *f*_0_, *E*_0_, and *S*_0_ represent the forces, cohesive energies, and stresses calculated using first-principles methods, respectively. Similarly, *f*, *E*, and *S* represent the corresponding values derived from the current EAM potential function. The terms ε*_j_*, ε*_k_*, and ε*_l_* are negligible and have no physical significance.

To optimize the parameters effectively, we first use the simulated annealing algorithm [47], a global optimization technique aimed at minimizing energy states. Subsequently, we apply a deterministic method, similar to conjugate gradients [48], to further refine the optimization process and achieve a more accurate local minimum.

**Figure 12:**
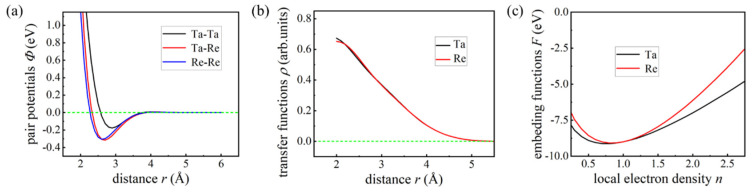
EAM potential function for Ta-Re alloy system: (**a**) pair potential functions, (**b**) transfer functions, (**c**) embedding functions.

Portability is critical when evaluating the practical application of the EAM potential function, and good portability relies on selecting appropriate reference structures. Enhancing transferability requires a larger number of reference configurations, but this introduces the risk of additional errors. Therefore, it is essential to balance the quantity of reference data with the accuracy of the model. In Equation (11), $w_{E}$ and $w_{s}$ are the weighting factors for cohesive energy and stress, respectively, and they play a significant role in the accuracy of force, energy, and stress fitting. Based on a series of test calculations, we determined that $w_{E}$ should be set to 20,000 and $w_{s}$ to 8000. The *Φ_αβ_*(*r*) with PPF, the *ρβ*(*r*) with TF, and the *F_α_*(*n*) with EF for each element serve as the target functions in the fitting process. In the Ta-Re alloy system, Figure 12 illustrates the fitted functions of our EAM potential model, with panels (a), (b), and (c) representing the PPF, TF, and EF, respectively. Notably, both the PPF and TF approach zero at atomic separations exceeding 6.0 Å, which led us to select a cutoff distance of 6.0 Å to terminate interactions between atoms in the Ta-Re alloy system. A comprehensive summary of all parameters obtained from the fitting process for the current EAM potential model is presented in Table 6.

### 4.4. Experimental Procedure

The Ta-Re binary alloy specimens were produced using a tungsten electrode arc furnace, with raw materials consisting of 99.99 wt% pure Re and Ta particles. The Re particles were obtained from Advanced Technology and Materials Co., Ltd. (Beijing, China), while the Ta particles were sourced from Ningxia Orient Tantalum Industry Co., Ltd. (Ningxia, China). To ensure uniformity, the Ta-Re alloy samples underwent five consecutive melting cycles (the elemental compositions of the Ta-Re alloys are shown in Table 7). Electromagnetic stirring was applied during the melting process, and the vacuum level in the tungsten electrode arc furnace was maintained at no less than 5 × 10^−3^ Pa.

X-ray diffraction (XRD) analysis was performed using a Shimadzu XRD 6000 diffractometer (Shimadzu Corporation, Kyoto, Japan) at room temperature. CuKα radiation, generated by a Cu X-ray tube operating at 15 mA and 30 kV, was used. The samples were scanned over a Bragg angle 2θ range from 20° to 90° at a rate of 2° per minute. Characterization was conducted with a JEOL SU3900 Scanning Electron Microscope (SEM) (JEOL, Kyoto, Japan) equipped with an Energy Dispersive Spectrometer (EDS), using an acceleration voltage of 20 kV. Nanoindentation tests were performed using a UMIS machine from Fischer-Cripps Laboratories, which is equipped with a Berkovich indenter and has a maximum load capacity of 400 mN.

## 5. Conclusions

In this study, we successfully developed the EAM potential function for Ta-Re alloys using the force-matching method. The bulk structure lattice constants and surface formation energies per unit area of Ta-Re alloys, as obtained from the potential function, are 12.196 Å, E_100_ = 0.16 × 10^−2^ eV, E_110_ = 0.10 × 10^−2^ eV, and E_111_ = 0.08 × 10^−2^ eV. The corresponding error values are 0.015 Å, 0.04 × 10^−2^ eV, 0.02 × 10^−2^ eV, and 0.01 × 10^−2^ eV, respectively, when compared with first-principles calculations. We also determined the atom-averaged binding energies for two cluster structures, Ta-rich (Ta_39_Re_20_) and Re-rich (Ta_20_Re_39_), with errors ranging from 1.64% to 1.98%. Ta-Re alloys with three compositions (Ta_48_Re_06_, Ta_30_Re_24_, Ta_06_Re_48_) were prepared, and their bulk moduli were measured. The experimentally measured bulk modulus values exhibited a trend of initially decreasing and then increasing, which was consistent with the trend observed in the calculated values. These results validate the accuracy and reliability of the EAM potential functions in characterizing various properties of Ta-Re alloy crystals, surfaces, defects, and clusters. The findings of this study provide valuable theoretical insights into the modification of properties in Ta/Re layered composites.

## Figures and Tables

**Table 1:** Surface formation energies per unit area for three different surfaces of Ta-Re alloys. (Units: eV/Å^2^).

*E*_surf_	*E*_100_	*E*_110_	*E*_111_
First-principles calculation	0.20 × 10^−2^	0.12 × 10^−2^	0.09 × 10^−2^
EAM	0.16 × 10^−2^	0.10 × 10^−2^	0.08 × 10^−2^

**Table 2:** Monovacancy defect formation energy for Ta_36_Re_18_ alloy bulk. (Units: eV).

Bulk	Ta1	Ta2	Ta3	Ta4	Ta5	Ta6	Ta7	Ta8	Ta9	Ta10
First-principles calculation	6.516	6.207	6.344	5.927	6.150	6.331	6.420	6.362	6.188	6.344
EAM	7.043	6.582	6.201	6.023	5.643	6.647	5.907	6.315	5.903	6.201
Bulk	Re1	Re2	Re3	Re4	Re5	Re6	Re7	Re8	Re9	Re10
First-principles calculation	6.783	6.751	6.732	6.935	6.251	7.306	6.911	7.082	6.320	6.344
EAM	6.145	6.296	6.897	7.291	6.743	7.279	6.845	6.753	6.465	6.291

**Table 3:** Average atomic binding energies for Ta_39_Re_20_ and Ta_20_Re_39_ clusters from first-principles calculation and EAM calculations, respectively. (Units: eV/atom).

System	First-Principles Calculation	EAM
Ta_39_Re_20_	10.681	10.893
Ta_20_Re_39_	10.708	10.884

**Table 4:** Bulk elastic moduli for different Ta and Re content mass ratios (unit: GPa).

Ta:Re	7.77:1	1.21:1	0.12:1
experimental value	77.31	66.67	145.27
calculated value	61.43	54.59	114.96

**Table 5:** List of non-equilibrium structures.

Structure Type	Number of Additions	Description	Configuration	Notes
Stress Structure	21	FCC compression and expansion	Ta_20_Re_12_	Modeling structural compression and expansion
Displacement Perturbation	4	Body-centered cubic, face-centered cubic, simple cubic, hexagonal perturbation	BCC, FCC, SC, HCP	Various perturbation types to simulate strain
Surface Structure	12	Low-index surfaces of BCC, FCC, SC, HCP structures	(100), (110), (111) planes	Focus on surface energy effects
Segregation Structure	10	Pure tantalum and rhenium in various configurations	BCC, FCC, SC, HCP, cluster configurations	Analyze segregation patterns at interfaces

**Table 6:** Parameters for the EAM potential function.

Pair potential functions											
Pair	C_1_		η_1_		C_2_	η_2_	k		φ		h
Ta-Ta	1.000		19.999		−71.074	4.538	2.621		4.304		3.423
Ta-Re	1.000		19.999		−100.023	4.653	2.031		0.000		3.423
Re-Re	1.000		19.999		−95.925	4.758	1.972		0.262		3.423
Transfer functions											
Element		a_1_		α		φ		β		h	
Ta		0.354		4.752		2.152		2.798		3.423	
Re		0.603		4.668		1.623		3.073		3.423	
Embedding functions											
Element			F_0_			γ			F_1_		
Ta			−10.000			0.573			1.000		
Re			−10.000			0.747			1.000		

**Table 7:** Calculation and experimental values of elemental contents in Ta-Re alloys.

		Ta_48_Re_06_	Ta_30_Re_24_	Ta_06_Re_48_
Calculate	Ta	88.8 at% (88.5 wt%)	55.5 at% (54.7 wt%)	11.2 at% (10.7 wt%)
	Re	11.2 at% (11.5 wt%)	44.5 at% (45.3 wt%)	88.8 at% (89.3 wt%)
Experiment	Ta	8.9 g (88.1 wt%)	5.5 g (55.0 wt%)	1.1 g (10.9 wt%)
	Re	1.2 g (11.9 wt%)	4.5 g (45.0 wt%)	9.0 g (89.1 wt%)

## Data Availability

All data in this paper have been included in the manuscript.

## Supplementary Materials

The following supporting information can be downloaded at: https://www.mdpi.com/article/10.3390/molecules29245963/s1, Figure S1. Dot plots for different Ta and Re content mass ratios (a) 7.77:1; (b) 1.21:1; (c) 0.12:1; Figure S2. Perturbation structure: (a) bcc, (b) fcc, (c) sc, (d) hcp. Among them, green spheres represent Ta atoms, while purple spheres denote Re atoms; Figure S3. Low index surfaces of structures: (a) bcc, (b) fcc, (c) sc, (d) hcp. Among them, green spheres represent Ta atoms, while purple spheres denote Re atoms; Figure S4. Pure tantalum and rhenium in various configurations: (a) bcc, (b) fcc, (c) sc, (d) hcp, (f) cluster. Among them, green spheres represent Ta atoms, while purple spheres denote Re atoms.
