# Supplementary material for: Construction and Experimental Validation of Embedded Potential Functions for Ta-Re Alloys
Source: Molecules. 2024 Dec 18;29(24):5963. doi: 10.3390/molecules29245963 (PMC11678435; doi:10.3390/molecules29245963)
Supplement: Supplementary file 1 [file molecules-29-05963-s001.zip › molecules-3342445-supplementary.pdf]

# Supplementary Materials for

# Construction and experimental validation of embedded potential functions for Ta-Re alloys

Figure S1

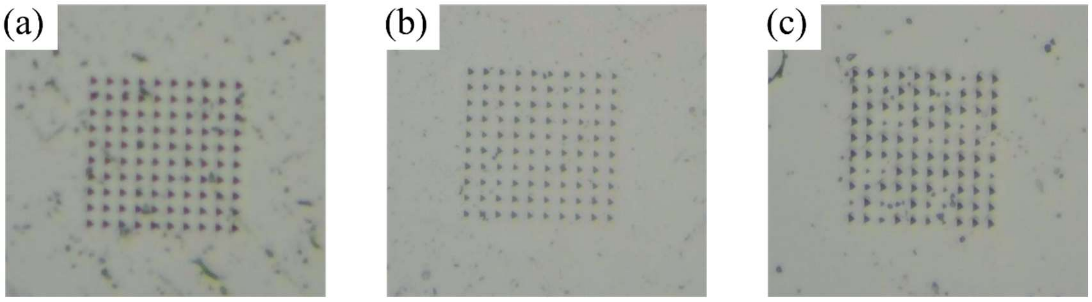


Figure S1. Dot plots for different Ta and Re content mass ratios (a)7.77:1; (b)1.21:1; (c)0.12:1

Below are the dot matrix data for different Ta and Re content mass ratios (a) 7.77:1; (b) 1.21:1; (c) 0.12:1

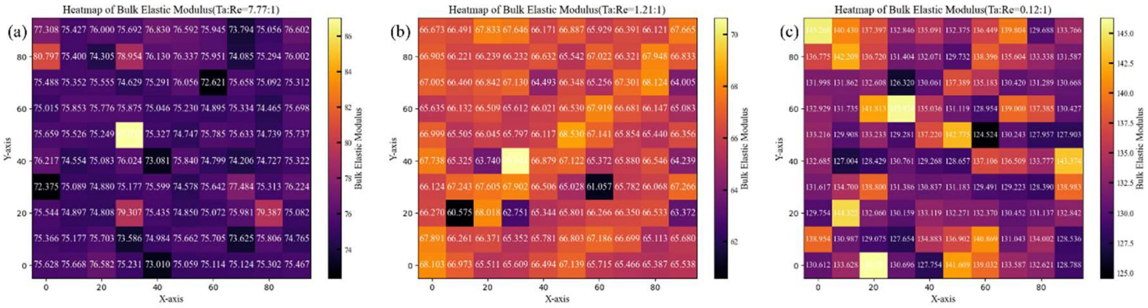


Figure S2

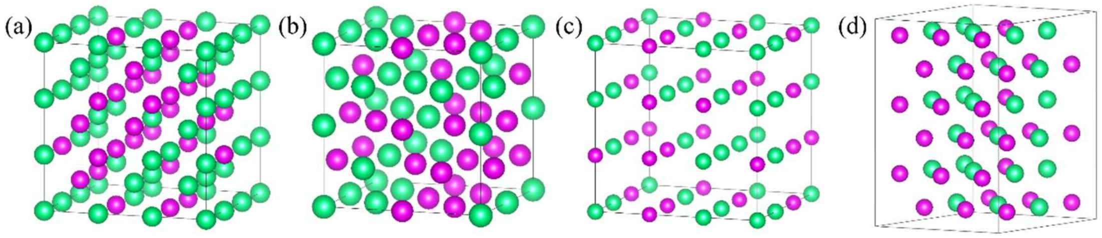


Figure S2. Perturbation structure: (a) bcc, (b) fcc, (c) sc, (d) hcp. Among them, green spheres represent Ta atoms, while purple spheres denote Re atoms.

Figure S3

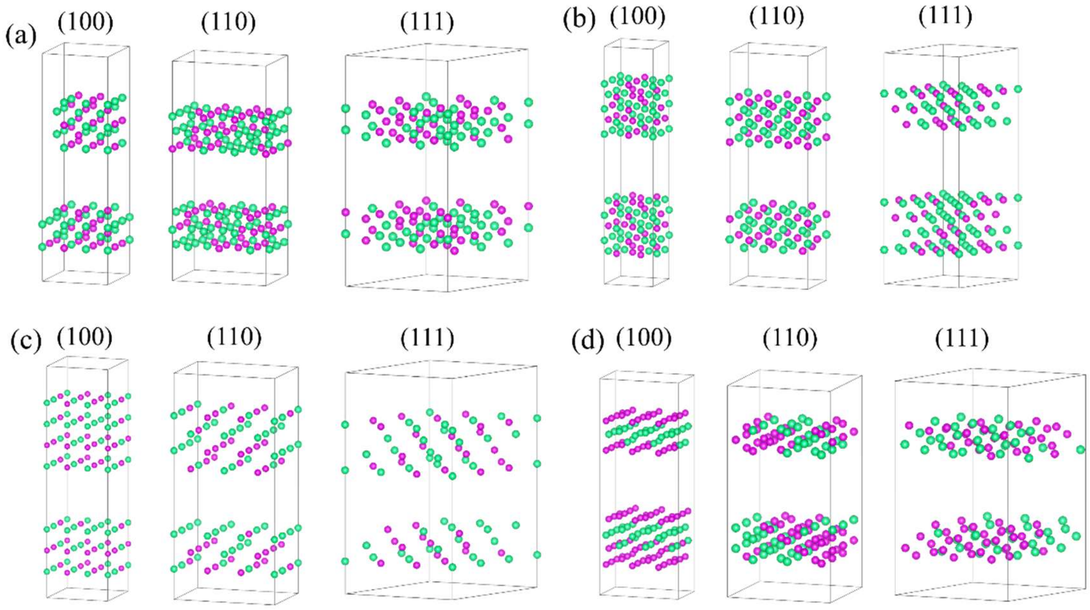


Figure S3. Low index surfaces of structures: (a) bcc, (b) fcc, (c) sc, (d) hcp. Among them, green spheres represent Ta atoms, while purple spheres denote Re atoms.

Figure S4

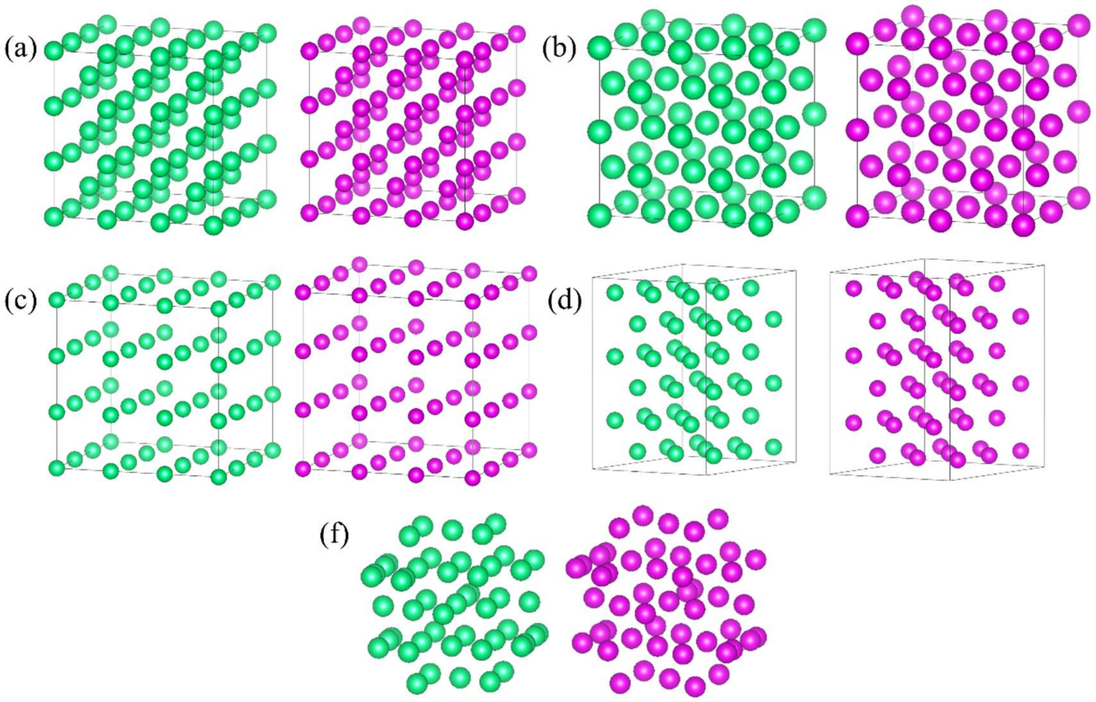


Figure S4. Pure tantalum and rhenium in various configurations: (a) bcc, (b) fcc, (c) sc, (d) hcp, (f) cluster. Among them, green spheres represent Ta atoms, while purple spheres denote Re atoms.
